# Association between cigarette smoking and colorectal cancer sidedness: A multi-center big-data platform-based analysis

**DOI:** 10.1186/s12967-021-02815-4

**Published:** 2021-04-15

**Authors:** Lu-Ping Yang, Zi-Xian Wang, Rui Zhang, Na Zhou, A-Man Wang, Wei Liang, Zhi-Qiang Wang, Hui-Yan Luo, Feng Wang, Ji-Wei Liu, Fang Liu, Xiao-Chun Zhang, Yun-Peng Liu, Ying Jin

**Affiliations:** 1 State Key Laboratory of Oncology in South China, Collaborative Innovation Center for Cancer Medicine, Sun Yat-Sen University Cancer Center, 651 Dong Feng Road East, Guangzhou, 510060 China; 2 Department of Colorectal Surgery, Cancer Hospital of China Medical University, Liaoning Cancer Hospital & Institute, Shenyang, 110042 China; 3 Precision Medicine Center of Oncology, The Affiliated Hospital of Qingdao University, No. 59 Haier Road, Qingdao, 266016 China; 4 Department of Oncology, The First Affiliated Hospital of Dalian Medical University, Dalian, 116011 China; 5 Department of Medical Oncology, The First Hospital of China Medical University, Shenyang, 110001 China; 6 Yiducloud Technology Ltd, Beijing, 100083 China

**Keywords:** Cigarette smoking, Colorectal cancer, Sidedness, Big-data platform, Prognosis

## Abstract

**Background:**

Sidedness (right/left) of colorectal cancer (CRC) is essential for treatment. Whether carcinogenesis of tobacco varies by sidedness remains unclear. The present study aims to evaluate the sidedness tendency of cigarette smoking and to explore its impact on prognosis.

**Methods:**

In the multi-center retrospective study, data on 46 166 Chinese CRC patients were extracted from a big-data platform. Logistic regression analyses were performed to evaluate qualitative and quantitative associations between smoking and tumor sidedness. Survival analyses were conducted in metastatic CRC.

**Results:**

History of smoking was associated with left-sided CRC (LSCRC; Adjusted odds ratio, 1.25; 95% CI, 1.16 − 1.34; *P* < .001). The sidedness tendency towards LSCRC increased from non-smokers, to ex-smokers, and to current smokers (*P* for trend < .001). Longer duration (*P* for trend < .001) and larger total amount of cigarette smoking (*P* for trend < .001) were more associated with LSCRC, respectively. The association was confirmed in both left-sided colon cancer and rectal cancer, but was stronger for rectal cancer (*P* = .016). Alcoholism significantly enhanced the association by 7% (*P* = .027). Furthermore, prognostic advantage of metastatic LSCRC diminished among ever-smokers, with contrary survival impacts of smoking on either side of CRC.

**Conclusions:**

History of smoking was associated with LSCRC in a positive dose–response relationship, and presented opposite prognostic impacts on right- and left-sided tumors. Smoking potentially plays an instrumental role in the mechanism for sidedness heterogeneity in CRC.

**Supplementary Information:**

The online version contains supplementary material available at 10.1186/s12967-021-02815-4.

## Introduction

Colorectal cancer (CRC) is the third most common cancer and its mortality ranks fourth worldwide [1, 2]. In China, the increasingly high incidence of CRC has roused intense attention, and the identification of etiologic and prognostic factors is an important task for oncologists.

Modifiable behaviors, including smoking, chronic alcohol intake, lack of physical exercise, and unhealthy diet, are risk factors for CRC [3, 4]. Previous studies have demonstrated a significant association of cigarette smoking with CRC incidence and mortality [5, 6]. Researchers have evaluated the association in subgroups of different primary tumor locations, and some proved it more solid for rectal cancer (RC) rather than colon cancer. This finding implied a differential susceptibility of carcinogenesis by cigarettes between subsites of colorectum. However, inconsistent conclusions were obtained from previous studies and none of them conducted a direct comparison between subsites. [6–10].

In recent years, the theory that right-sided colon cancer (RSCC) and left-sided colorectal cancer (LSCRC) are biologically heterogeneous has been established and widely accepted, but the underlying mechanism has not been clear yet [11]. We wondered whether smoking-related cancer susceptibility might vary between RSCC and LSCRC, and consequently result in the difference of tumor sidedness tendency between ever and never smokers and in the heterogeneity of molecular features between sidedness. It might also play a part in the prognostic advantage of LSCRC over RSCC among metastatic CRC (mCRC) [12, 13].

In this retrospective study, we evaluated the relationship between cigarette smoking and CRC sidedness based on a multi-center big-data intelligence platform with the aim of exploring potential tendencies for sidedness related to cigarette smoking, and assessed its potential prognostic influence regarding tumor sidedness.

## Methods

### Data source and study population

All data used in the study were extracted from the Bigdata Alliance for Colorectal Cancer (BACC) platform (YiduCloud Technology Ltd., Beijing, China) which is a big-data intelligence platform that integrates and converges mass multi-source heterogeneous electronic health-records data from multiple medical centers or hospitals all over China.

A total of 71 429 Chinese CRC patients from the BACC platform diagnosed from January 1, 2000 to April 30, 2018 comprised the study population. The inclusion criteria were as follows: (i) the patient was diagnosed with colorectal adenocarcinoma by histopathology and (ii) was ≥ 18 years old. The exclusion criteria were as follows: (i) information for confirming CRC sidedness was unavailable; (ii) information about the patient’s history of smoking, alcohol or familial malignancy was unavailable. Additional file 1: Figure S1 shows the flowchart of the patient selection process. The final cohort for the analyses consisted of 46 166 patients.

The study was approved by the Institute Research Ethics Committee at the Sun Yat-sen University Cancer Center (ID: B2018-154–01), and was conducted in accordance with best clinical practices and the Declaration of Helsinki. All authors had access to the study data and reviewed and approved the final manuscript.

### Definitions of the study variables

History of smoking, alcohol intake, and familial malignancy were extracted from the electronic medical records and structured in the BACC platform. Patients who reported a history of smoking were identified as ever-smokers; those who had quit smoking for ≥ 3 months were classified as ex-smokers and the rest ever-smokers were identified as current smokers. The quantification of cigarette smoking was measured in terms of daily consumption (i.e., the number of cigarettes smoked per day), duration (years) of smoking, and a smoking index which is the product of the former two measurements (cigarettes * years). The cut-off points for the number of cigarettes smoked per day and the duration (years) of smoking were adopted from a previous study [14], and the median value of the smoking index was used as its cut-off point.

The primary tumor location was defined in accordance to the diagnosis recorded in the BACC platform, and was used to clarify the definition of sidedness. RSCC was defined as a tumor in the cecum, ascending colon, or transverse colon. LSCRC was defined as a tumor located from the splenic flexure to the rectum. Left-sided colon cancer (LSCC) was defined as LSCRC excluding RC.

### Statistical analysis

Categorical variables were analyzed using the Chi-square test. Temporal trends in the prevalence of smoking were evaluated using the Cochran-Armitage trend test. The associations between cigarette smoking and CRC sidedness were examined using binary logistic regression. In the logistic regression, we labelled RSCC as the reference. The odds ratios (OR) and 95% CIs were estimated after adjustments for age at diagnosis, sex, and family history of malignancy, which have been reported to be potential confounders[15]. Multicollinearity of the regression models was determined by variance inflation factor (VIF); a VIF ≤ 10 indicates a robust model with insignificant influence by collinearity. Given the stronger carcinogenic impact of smoking on the rectum, [5] we stratified LSCRC to LSCC and RC in the correlation analyses. We conducted subgroup analyses in subsets of patients by age (18 − 39/40 − 59/60 − 79/ ≥ 80 years), sex (males/females), year of diagnosis (2000 − 2011/2012 − 2014/2015 − 2018), family history (yes/no), history of chronic alcohol intake (yes/no), and metastatic status at initial diagnosis (M1/M0). The ORs were compared in binary subgroups on the log scale. [16].

We further explored the prognostic effects of smoking and tumor sidedness on mCRC. The survival analyses were restricted to the patients with mCRC whose follow-ups lasted 3 months or longer (cohort M, n = 2 272), using Kaplan–Meier method with log-rank test for comparisons of overall survival (OS). Multivariate Cox analyses including variables with *P*-values < 0.05 in univariate Cox analyses were also conducted.

All statistical analyses were performed using SPSS Statistics Version 22.0 (IBM Corp, Armonk, NY), R Version 3.5.3 (The R Foundation, Vienna, Austria), and GraphPad Prism Version Version 7.04 (GraphPad Software, San Diego, CA). A two-tailed *P*-value < 0.05 was considered statistically significant.

## Results

### Characteristics of the study population

Demographic and clinical characteristics of the study cohort are shown in Table 1. Among the 46 166 eligible patients, the median age at diagnosis was 61 years (range, 18 − 102) and 60.4% were males. Nearly half (46.3%) of the cases were diagnosed in the past three years. The majority (63.3%) of the tumors were located in the rectum, followed by 22.8% with LSCC and 13.8% with RSCC.

**Table 1:** Demographic and clinical characteristics of the study population and cohorts according to cigarette and alcohol intake status

Variable	All cases (N = 46,166)	Cigarette smoking status		
		Ever (n = 10,712)	Never (n = 35,454)	*P* value
Age, years				.14 ^a^
18−39	2359 (5.11%)	371 (3.46%)	1988 (5.61%)	
40−59	18,028 (39.05%)	4438 (41.43%)	13,590 (38.33%)	
60−79	23,297 (50.46%)	5504 (51.38%)	17,793 (50.19%)	
≥ 80	2482 (5.38%)	399 (3.72%)	2083 (5.88%)	
Sex				< .001
Male	27,869 (60.37%)	10,202 (95.24%)	17,667 (49.83%)	
Female	18,297 (39.63%)	510 (4.76%)	17,787 (50.17%)	
Year of diagnosis				< .001
2000−2011	5962 (12.91%)	1131 (10.56%)	4831 (13.63%)	
2012−2014	18,822 (40.77%)	4274 (39.90%)	14,548 (41.03%)	
2015−2018	21,382 (46.32%)	5307 (49.54%)	16,075 (45.34%)	
Primary tumor location				< .001
Cecum, ascending and transverse colon cancer	6393 (13.85%)	1175 (10.97%)	5218 (14.72%)	
Splenic flexure, descending and sigmoid colon cancer	10,548 (22.85%)	2378 (22.20%)	8170 (23.04%)	
Rectum	29,225 (63.30%)	7159 (66.83%)	22,066 (62.24%)	
Primary tumor sidedness				< .001
Right-sided	6393 (13.85%)	1175 (10.97%)	5218 (14.72%)	
Left-sided	39,773 (86.15%)	9537 (89.03%)	30,236 (85.28%)	
Alcohol intake history				< .001
No	37,819 (81.92%)	4701 (43.89%)	33,118 (93.41%)	
Yes	8347 (18.08%)	6011 (56.11%)	2336 (6.59%)	
Metastasis at diagnosis				< .001
No	15,523 (33.62%)	3440 (32.11%)	12,083 (34.08%)	
Yes	8567 (18.56%)	1889 (17.63%)	6678 (18.84%)	
Unknown	22,076 (47.82%)	5383 (50.25%)	16,693 (47.08%)	
Family history				< .001
Yes	4264 (9.24%)	1256 (11.73%)	3008 (8.48%)	
No	41,902 (90.76%)	9456 (88.27%)	32,446 (91.52%)	

^a^*P* value of student’s t test to compare means

**Fig. 1:**
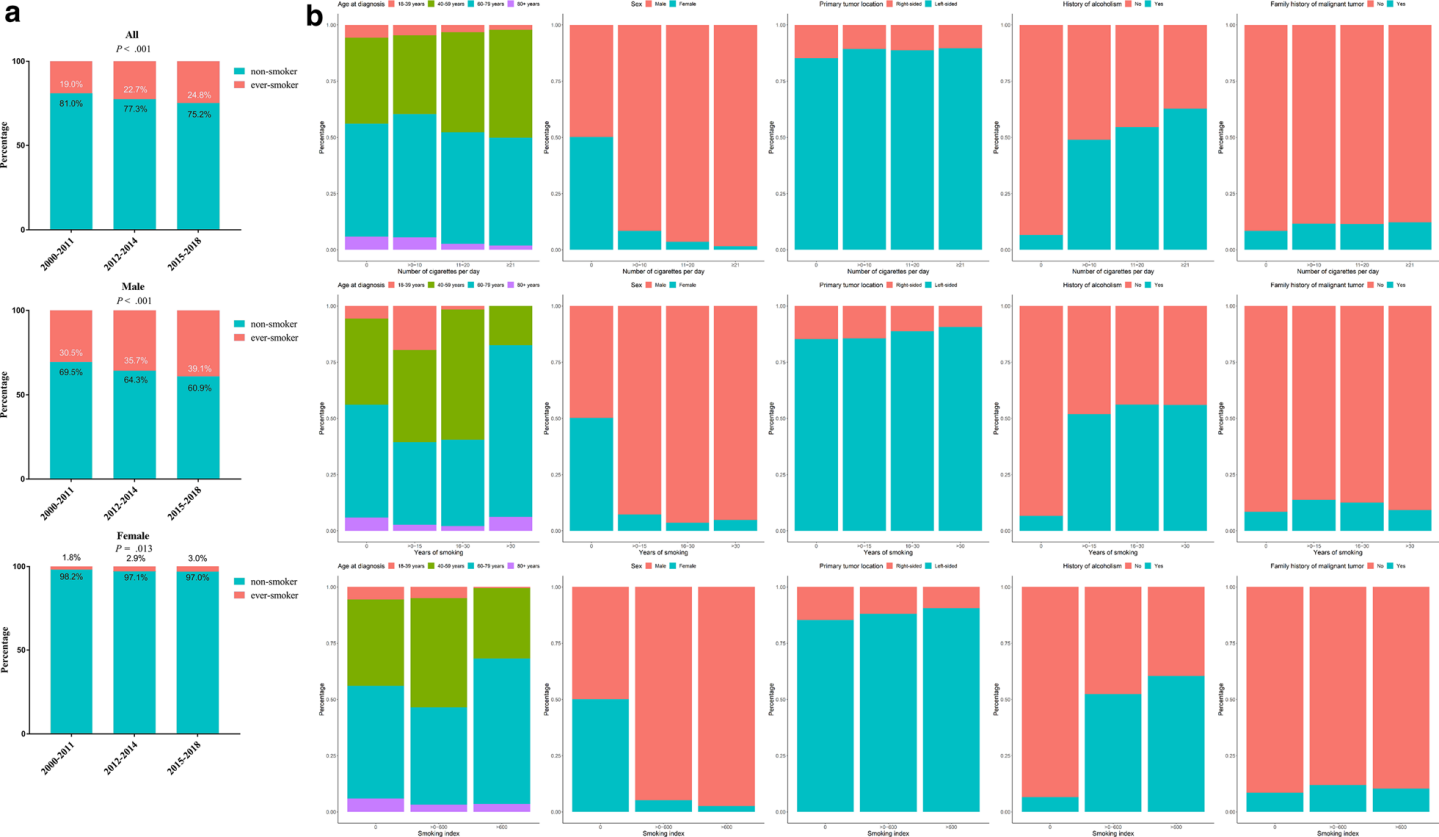
Stacked bar chart of clinical features of the study cohort a, temporal trends for the proportions of smokers among patients with colorectal cancer; b, the composition of patients regarding age, sex, primary tumor location, history of alcoholism and family history of malignant tumors stratified by daily consumption, years of duration, and total amount of cigarette smoking, respectively

This study consisted of 10 712 (23.2%) ever-smokers and 35 454 (76.8%) non-smokers. They were diagnosed at a similar age (*P* = 0.140), but a noticeable gender imbalance was found among the ever-smokers of whom 95.2% were males. More than half of the smokers exhibited concurrent alcoholism. A statistically significant increase was found among patients with CRC who smoked (from 19.0% in 2000–2011 to 24.8% in 2015–2018) in both genders (Fig. 1a). Females showed a more pronounced increase in the magnitude of smokers (by 66.7%). The smoking trends implied that except bans on public indoor smoking, cigarette smoking remained a crucial public health issue. In terms of clinical features of patients with different tobacco-consuming amount, we observed that the proportion of elderly, male patients or patients with LSCRC, history of chronic alcoholic intake or family history of cancer tended to grow as the increment of cigarettes smoked (Fig. 1b).

### Sidedness tendency

Table 2 shows the odds ratios of the sidedness tendency towards LSCRC over RSCC by smoking status. A history of smoking was found to be associated with LSCRC (Adjusted odds ratio [AOR], 1.25; 95% CI, 1.16 − 1.34; *P* < 0.001). Patients who quit smoking showed no tendency towards either side, and current smokers showed a significant tendency towards LSCRC (*P* for trend < 0.001). Moreover, a stronger dose–response relationship was found for a longer duration of smoking (*P* for trend < 0.001) and a larger smoking index (*P* for trend < 0.001) with LSCRC. Among heavy smokers with a smoking history > 30 years or a smoking index > 600, the likelihood to develop LSCRC other than RSCC was respectively, 55% and 46% higher than for non-smokers. However, a higher number of cigarettes smoked per day did not indicate a stronger tendency towards LSCRC. These findings present a trend in the long-term effect of cigarette smoking on CRC sidedness. No multicollinearity was observed to confound the association (See Additional file 2 Table S1). We further adjusted for the geographic location of medical centers and observed consistent results (See Additional file 2 Table S2).

**Table 2:** Adjusted odds ratios for sidedness of colorectal cancer (left-sided vs. right-sided) according to smoking history

	No. of cases	AOR (95%CI) ^a^	*P* for trend ^b^
Cigarette smoking			
Never	30,236/5218	Reference	< .001
Ever	9537/1175	1.25 (1.16, 1.34)	
Current smoking status			
Non-smokers	30,236/5218	Reference	< .001
Ex-smokers	2570/287	0.90 (0.80, 1.02)	
Current smokers	2215/388	1.39 (1.22, 1.58)	
Unknown cessation	4752/500	1.43 (1.29, 1.58)	
Number of cigarettes per day			
0	30,236/5218	Reference	.790
> 0−10	2178/259	1.32 (1.16, 1.52)	
11−20	4382/557	1.19 (1.08, 1.32)	
≥ 21	1568/181	1.30 (1.11, 1.53)	
Unknown	1409/178	1.24 (1.06, 1.46)	
Duration of smoking (years)			
0	30,236/5218	Reference	< .001
> 0−15	1025/173	0.98 (0.83, 1.16)	
16−30	4349/551	1.18 (1.07, 1.30)	
> 30	3016/310	1.55 (1.36, 1.76)	
Unknown	1147/141	1.27 (1.06, 1.52)	
Smoking index			
0	30,236/5218	Reference	< .001
> 0−600	4883/657	1.14 (1.04, 1.25)	
> 600	2777/289	1.46 (1.28, 1.66)	
Unknown	1877/229	1.29 (1.12, 1.49)	

^a^After adjustment for age, sex, and family history

^b^*P* value was evaluated based on the cases without unknown data

The multivariate analysis showed that females tended to develop RSCC (AOR, 0.78; 95% CI, 0.73 − 0.83; *P* < 0.001), as did patients with a family history of malignant tumors (AOR, 0.64; 95% CI, 0.59 − 0.69; *P* < 0.001). Furthermore, there was no significant association between a history of alcohol consumption and sidedness of CRC (AOR, 1.06; 95% CI, 0.96 − 1.15; *P* = 0.110). However, the sidedness tendency of cigarette smoking tended to grow stronger from never smokers, non-drinker smokers, to drinker-smokers (AOR for non-drinker smokers, 1.21; 95% CI, 1.09 − 1.33; AOR for drinker-smokers, 1.28; 95% CI, 1.17 − 1.41; *P* for trend < 0.001). Additional file 2: Table S3 presents the distribution of clinical variables across the tumor sidedness.

### Sidedness tendency stratified by intestinal segment

**Fig. 2:**
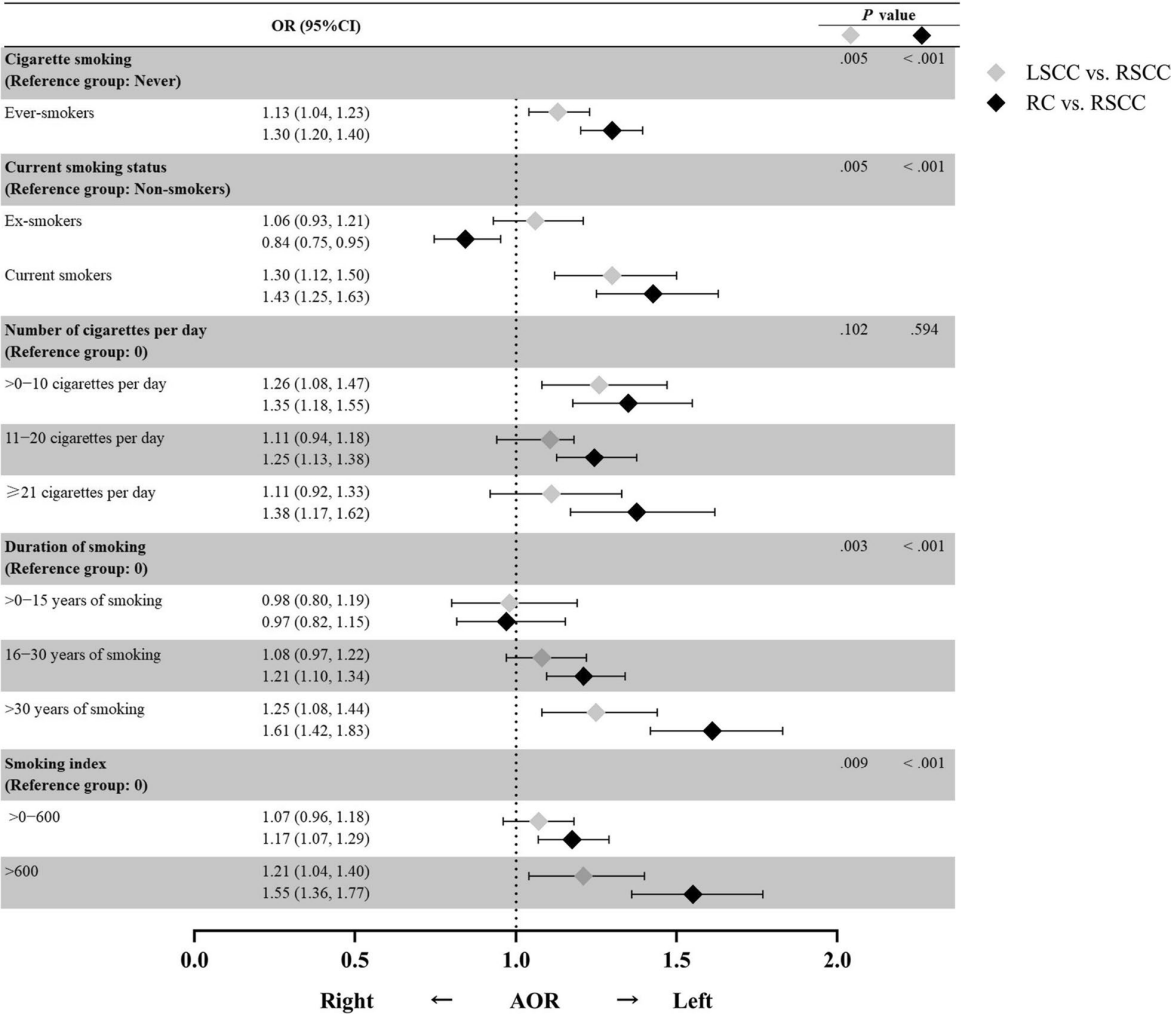
Associations of history of smoking with left-sided colon cancer and rectal cancer over right-sided colon cancer. *RSCC* right-sided colon cancer, *LSCRC* left-sided colorectal cancer

Among the patients diagnosed with LSCRC, the primary tumor for 10 548 (26.5%) was located in the colon, and for 29 225 (73.5%) it was located in the rectum. Smokers tended to have LSCC rather than RSCC (AOR, 1.13; 95% CI, 1.04 − 1.23; *P* = 0.010), and to have RC rather than RSCC (AOR, 1.30; 95% CI, 1.20 − 1.40; *P* < 0.001). Both intestinal segments showed solid associations with a history of smoking, while a significantly stronger tendency towards sidedness was found in RC (*P* = 0.016). Similar results were obtained in the dose–response analyses (Fig. 2). As the duration and total amount of cigarette consumption increased, gaps between the associating strengths of LSCC and RC became wider, indicating that the sidedness tendency towards RC was more sensitive to long-term dose accumulation of tobacco compared with that towards LSCC. Smoking cessation did not significantly reverse the trend in sidedness for the patients with colon cancer; however, ex-smokers were more likely to develop RSCC than RC (AOR, 0.84; 95% CI, 0.75 − 0.95).

### Subgroup analyses of sidedness tendencies

**Fig. 3:**
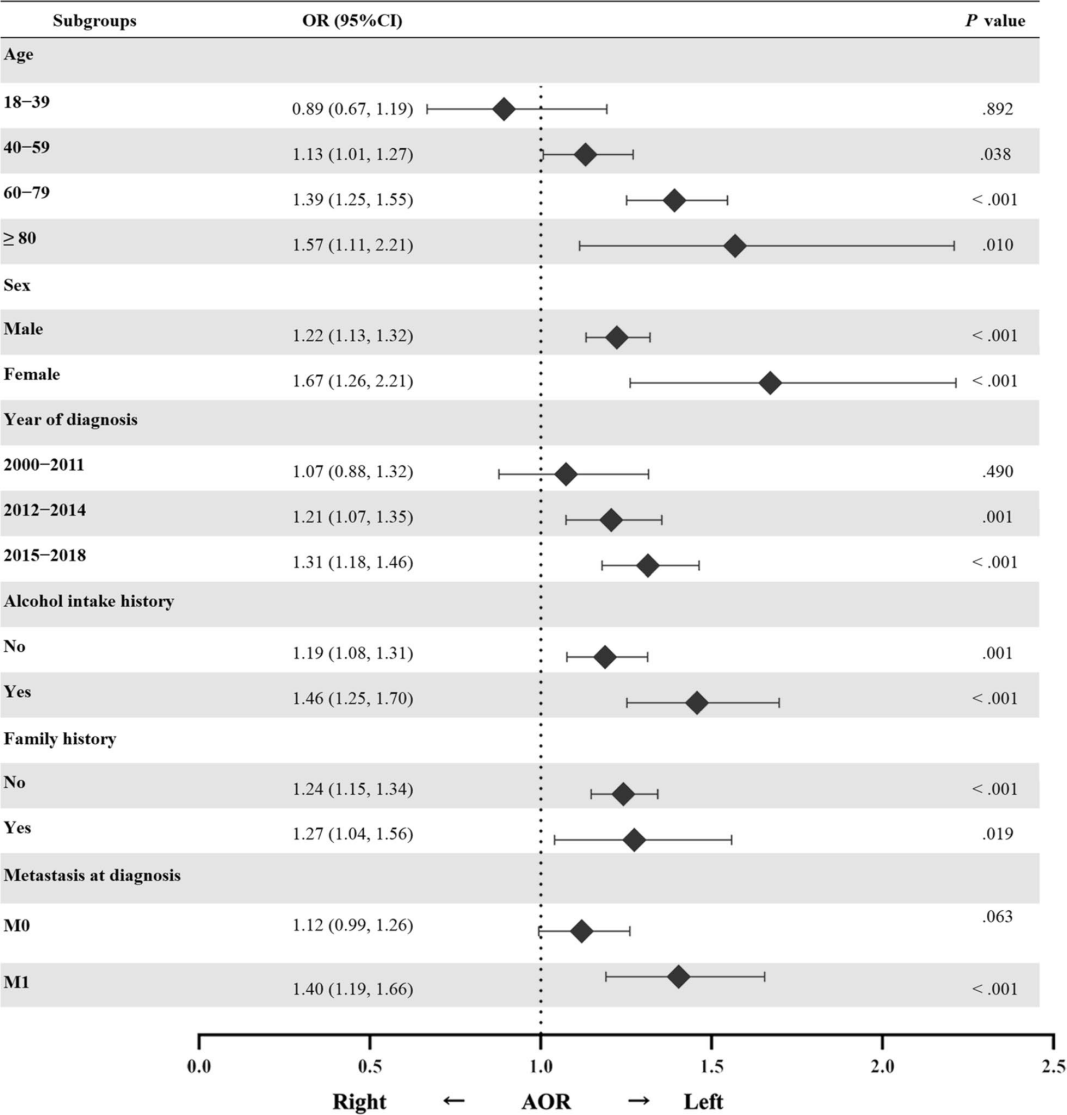
Subgroup analyses for associations of history of smoking with left-sided colorectal cancer over right-sided colon cancer

Chronic alcohol intake, which is usually concurrent with a habit of smoking, significantly enhanced the sidedness tendency towards LSCRC (*P* = 0.027). Among the patients diagnosed at  ≥ 40 years old, a significant association was found between cigarette smoking and LSCRC, which tended to increase with age; however, this tendency was attenuated among the younger patients (aged 18 to 39 years), possibly due to inadequate accumulation of cigarettes. The sidedness tendency was robust in the subgroups classified by sex, history of alcohol intake, or family history of malignant cancer. Figure 3 shows the AORs for LSCRC compared to RSCC in these subgroups.

### Prognostic impact of sidedness influenced by smoking status

**Fig. 4:**
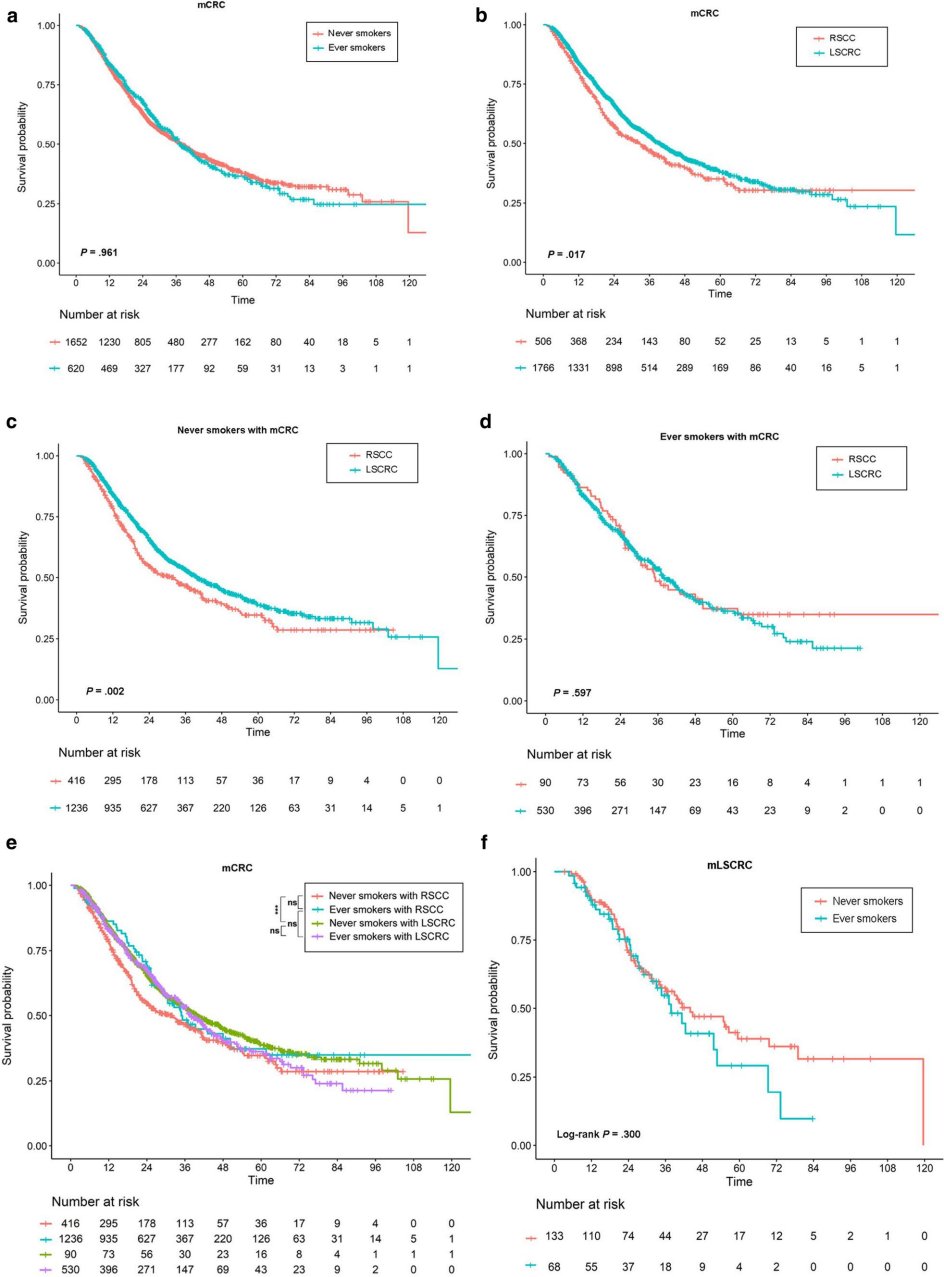
Survival curves of patients with metastatic colorectal cancer stratified by tumor sidedness and history of smoking. a-b, survival curves of cohort M stratified by history of smoking (**a**) or by tumor sidedness (**b**); **c-d**, survival curves of never smokers (**c**) or ever-smokers (**d**) stratified by tumor sidedness; **e**, comparisons of overall survival regarding tumor sidedness and history of smoking; **f**, survival curves of patients with metastatic LSCRC treated with cetuximab stratified by history of smoking.* mCRC* metastatic colorectal cancer, *RSCC* right-sided colon cancer, *LSCRC* left-sided colorectal cancer. ***stands for < .001; *ns* stands for not significant. *CRC* colorectal cancer

Since OS differed between metastatic LSCRC and RSCC, the prognostic impact of cigarette smoking on heterogeneity of sidedness in mCRC was in need of clarification. The median follow-up duration of cohort M (n = 2 272) was 31.12 months, and the clinical features are shown in Additional file 2: Table S4. Cigarette smoking was not proven to be an independently prognostic factor (Hazard ratio [HR], 1.00; 95% CI, 0.87 − 1.14; *P* = 0.961; See Fig. 4a). The prognosis of patients with LSCRC was significantly more favorable (HR, 0.85; 95% CI, 0.74 − 0.97; *P* = 0.017; See Fig. 4b), which was consistent with previous researches. Similar results were obtained in non-smokers in that they had a better prognosis for LSCRC over RSCC (HR, 0.79; 95% CI, 0.67 − 0.92; *P* = 0.002; See Fig. 4c), while the prognostic advantage of LSCRC was eliminated among the ever-smokers (HR, 1.09; 95% CI, 0.80 − 1.49; *P* = 0.597; See Fig. 4d). An interesting result was observed and might be the underlying reason, that is, a history of smoking relatively elongated the median OS of patients with RSCC by 2.7 months and on the contrary shortened that of patients with LSCRC by 6.1 months (although not statistically significant; See Fig. 4e and Additional file 2: Table S5). For patients with LSCRC, heavy smokers with smoking index > 600 showed marginally poorer prognosis (HR, 1.22; 95% CI, 0.99 − 1.49; *P* = 0.059); however, numerically lower HR was observed in RSCC (See Additional file 2: Table S6). It implied that cigarette smoking was potentially a favorable prognostic factor for RSCC and an unfavorable factor for LSCRC. Furthermore, we observed an insignificant reduce of OS by smoking in patients with metastatic LSCRC who received cetuximab (See Fig. 4f). It could not explain the loss of prognostic advantage for metastatic LSCRC and further investigation was needed.

## Discussion

In recent years, concerns have been raised that sidedness of colorectal cancer could influence biological behaviors of the tumor [11, 12, 17]. The difference in etiology might be the underlying mechanism of the heterogeneity between RSCC and LSCRC. Cigarette smoking, as a risk factor for mortality of CRC introducing genetic alterations and epigenetic imprints [18, 19], has the possibility to attribute to the heterogeneity. Thus, we evaluated the hypothesis in the present study based on 46 166 Chinese patients from the multi-center BACC platform, and proved cigarette smoking to have a positive dose–response relationship with LSCRC. A 2006 study by Zissman et al. reported an association between tobacco use and distal CRC [8], which was consistent with the results of our study; however, it lacked the analysis of proximal CRC, and the information has not been updated in recent years and was not confirmed in Asian populations. Liang et al. found a higher risk of developing cancer in the rectum than the colon among smokers, [6] but there were three studies showed inconsistent findings [7, 9, 10]. These results were controversial, and also raised the concern that the sidedness tendency towards LSCRC was, by nature, towards RC, not LSCC. The inconsistent results in terms of colon cancer subsites might be attributed to the different sample sizes and analytic methods. Those previous studies conducted indirect comparisons of CRC risks including healthy populations. In the present study, we conducted a direct comparison among patients in a large population from multiple medical centers; the dose-relationship analyses improved the credibility of our findings. Moreover, we stratified LSCRC to LSCC and RC and validated the association respectively.

Hecht reported the presence of sixty carcinogens in cigarette smoke and more than sixteen in unburned tobacco, among which tobacco-specific nitrosamines, polycyclic aromatic hydrocarbons and aromatic amines possibly play a key role in carcinogenesis [18]. Analyses of corresponding genetic alterations have found cigarette smoking was in relation to MSI-H, CIMP-H, BRAF mutation, p53 mutation, APC mutation, and promoter 1A methylation in CRC [19–21]. The association of CRC sidedness with cigarette smoking which we observed in this study supported the hypothesis that tobacco has more intensive carcinogenic effects on LSCRC, and consequently, leave specific tobacco-related molecular signatures in LSCRC different from RSCC. LSCRC has been found to be associated with CIN, p53 mutation and NRAS mutation over RSCC [22–24]. Therefore, p53 mutation might be the molecular feature left by tobacco tending to LSCRC. Further bioinformatic studies are needed to uncover specific mechanisms behind tumorigenesis.

The impact of cigarette smoking on prognosis or response to therapy of CRC was not clear yet. We explored it among patients with mCRC since left-sided tumor location has been proved a favorable prognostic indicator [17, 25]. However, in the present study, a history of smoking was neither a positive nor a negative prognostic factor of mCRC, and it even relatively prolonged OS among patients with metastatic RSCC and diminished the prognostic advantage of LSCRC, which was a novel discovery and had not been reported before. Meanwhile, we observed numerically-shortened OS in smokers with LSCRC and thus speculated smoking might reduce the efficacy of the targeted drug specially for LSCRC, cetuximab, for instance. We found comparable efficacy of cetuximab between ever-smokers and never-smokers with LSCRC; previous studies suggested that smoking habit might suppress survival benefit from second-line cetuximab-based treatment, though [26]. In other types of cancer, there was evidence that cigarette smoking was not unfavorable for prognosis as presumed [27–30]. Smoking could even protect patients with apolipoprotein E positive breast cancer from anticancer-treatment-induced cognitive impairment. Several researches reported that smoking had potentials to improve efficacy of immunotherapy among patients with non-small-cell lung cancer, probably by introducing neoantigens, increasing tumor mutation burden, or upregulating PD-L1 expression. [31, 32]. All these findings implied that despite carcinogenesis by tobacco, a history of smoking did not necessarily indicate a worse prognosis or less efficacy of treatment. This might lighten the mental burden of cancer patients who attributed smoking to the mortality and suffered from self-accusation. Nevertheless, smoking cessation was strongly recommended [32]. On the other hand, the different influences of smoking on survival between sidedness (See Additional file 2: Table S6) also suggested that tobacco alongwith its products might have heterogeneous biological effects in right- and left-sided tumors.

To the best of our knowledge, this is the first study to investigate the association between cigarette smoking and CRC sidedness based on a large population. On one hand, these findings suggest novel epidemiological features of CRC and its sidedness; on the other hand, new evidence is provided that biological heterogeneity could be partially caused by imbalanced carcinogenic effects of smoking between RSCC and LSCRC.

The present study has several limitations. First, information about cigarette -smoking history was collected through enquiry during the patients’ first admission, and might have recall bias and inaccuracies, especially for numerical information. To diminish these effects, we converted continuous variables into categorical ones. Second, incomplete follow-up information limited larger scale survival analyses. Updated follow-ups and cohort expansion are needed for further validation. Third, molecular features were not included in the analyses due to limited data available; studies should investigate possible molecular mechanisms in the future.

In conclusion, among patients with CRC, a history of cigarette smoking was associated with LSCRC in a positive dose–response relationship, and was largely dependent on long-term accumulation. Smoking cessation weakened the association while history of concurrent alcoholism could enhance it. Smoking was not unfavorable for prognosis and it showed varied tendencies of hazards for right- and left-sided tumors. Therefore, cigarette smoking might be instrumental in the mechanism for heterogeneity of sidedness.

## Supplementary Information

**Additional file 1: Figure S1.** Flowchart of the patient selection process.

**Additional file 2: Table S1.** Assessment of multicollinearity of the regression models for the association analyses. **Table S2.** Odds ratios for sidedness of colorectal cancer according to smoking history adjusted for age, sex, family history and geographic location of medical centers. **Table S3.** Clinical features of the study cohort stratified by tumor sidedness. **Table S4.** Demographic and clinical characteristics of the cohort M (n = 2 272). **Table S5.** Survival statistics of patients with metastatic colorectal cancer tumor regarding sidedness and history of smoking (n = 2 272). **Table S6.** Death risks of cohort M regarding cigarette smoking on a quantitation basis.

## Data Availability

The key raw data have been deposited into the Research Data Deposit (http://www.researchdata.org.cn) with the Approval Number of RDDA2020001769. The data analyzed during the current study are available from the corresponding author on reasonable request.

## Abbreviations

BACC: Bigdata Alliance for Colorectal Cancer
CI: Confidence interval
CRC: Colorectal cancer
HR: Hazard ratio
LSCC: Left-sided colon cancer
LSCRC: Left-sided colorectal cancer
mCRC: Metastatic colorectal cancer
OR: Odds ratios
OS: Overall survival
RC: Rectal cancer
RSCC: Right-sided colon cancer
VIF: Variance inflation factor
