# Supplementary figures and images for: Association between cigarette smoking and colorectal cancer sidedness: A multi-center big-data platform-based analysis
Source: J Transl Med. 2021 Apr 15;19:150. doi: 10.1186/s12967-021-02815-4 (PMC8048178; doi:10.1186/s12967-021-02815-4)

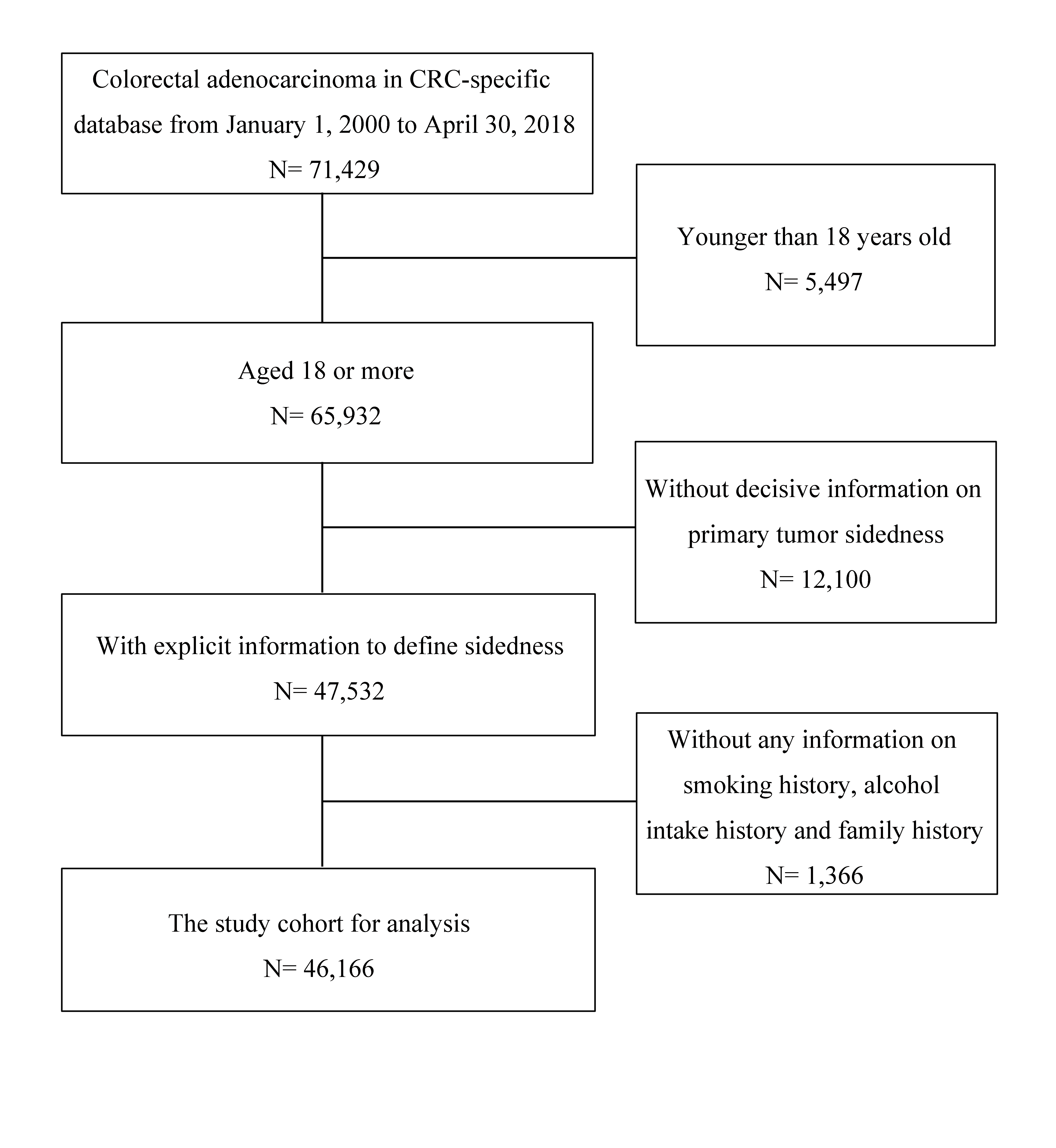

Supplement: Supplementary file 1 — Additional file 1: Figure S1. Flowchart of the patient selection process. [file 12967_2021_2815_MOESM1_ESM.tif]
