# Supplementary material for: Association between cigarette smoking and colorectal cancer sidedness: A multi-center big-data platform-based analysis
Source: J Transl Med. 2021 Apr 15;19:150. doi: 10.1186/s12967-021-02815-4 (PMC8048178; doi:10.1186/s12967-021-02815-4)
Supplement: Supplementary file 2 — Additional file 2: Table S1. Assessment of multicollinearity of the regression models for the association analyses. Table S2. Odds ratios for sidedness of colorectal cancer according to smoking history adjusted for age, sex, family history and geographic location of medical centers. Table S3. Clinical features of the study cohort stratified by tumor sidedness. Table S4. Demographic and clinical characteristics of the cohort M (n = 2 272). Table S5. Survival statistics of patients with metastatic colorectal cancer tumor regarding sidedness and history of smoking (n = 2 272). Table S6. Death risks of cohort M regarding cigarette smoking on a quantitation basis. [file 12967_2021_2815_MOESM2_ESM.docx]

**Supplementary Table 1. Assessment of multicollinearity of the regression models for the association analyses**

| **Analyzing factor** | **VIF** |
| --- | --- |
| Cigarette smoking | 1.185 |
| Current smoking status | 1.095 |
| Number of cigarettes per day | 1.085 |
| Duration of smoking (years) | 1.096 |
| Smoking index | 1.064 |

Abbreviations: VIF, variance inflation factor.

**Supplementary Table 2. Odds ratios for sidedness of colorectal cancer according to smoking history adjusted for age, sex, family history and geographic location of medical centers**

|  | **AOR (95%CI) ^a^** | ***P* for trend** **^b^** |
| --- | --- | --- |
| **Cigarette smoking** |  |  |
| Never | Reference | < .001 |
| Ever | 1.28 (1.19, 1.38) |  |
| **Current smoking status** |  |  |
| Non-smokers | Reference | < .001 |
| Ex-smokers | 1.20 (1.07, 1.36) |  |
| Current smokers | 1.46 (1.28, 1.67) |  |
| Unknown cessation | 1.24 (1.12, 1.38) |  |
| **Number of cigarettes per day** |  |  |
| 0 | Reference | .079 |
| >0−10 | 1.32 (1.15, 1.50) |  |
| 11−20 | 1.22 (1.10, 1.35) |  |
| ≥21 | 1.34 (1.14, 1.57) |  |
| Unknown | 1.38 (1.17, 1.62) |  |
| **Duration of smoking (years)** |  |  |
| 0 | Reference | < .001 |
| >0−15 | 1.02 (0.86, 1.21) |  |
| 16−30 | 1.21 (1.09, 1.34) |  |
| >30 | 1.52 (1.34, 1.73) |  |
| Unknown | 1.36 (1.13, 1.62) |  |
| **Smoking index** |  |  |
| 0 | Reference | < .001 |
| >0−600 | 1.16 (1.06, 1.27) |  |
| >600 | 1.48 (1.30, 1.69) |  |
| Unknown | 1.39 (1.21, 1.61) |  |

^a^ After adjustment for age, sex, family history and geographic location of medical center.

^b^ *P* value was evaluated based on the cases without unknown data.

**Supplementary Table 3. Clinical features of the study cohort stratified by tumor sidedness**

| **Variable** | **Right-sided** | **Left-sided** |
| --- | --- | --- |
| **Age, years** |  |  |
| 18−39 | 458 (7.2%) | 1901 (4.8%) |
| 40−59 | 2322 (36.3%) | 15706 (39.5%) |
| 60−79 | 3194 (50.0%) | 20103 (50.5%) |
| ≥ 80 | 419 (6.6%) | 2063 (5.2%) |
| **Sex** |  |  |
| Male | 3429 (53.6%) | 24440 (61.4%) |
| Female | 2964 (46.4%) | 15333 (38.6%) |
| **Year of diagnosis** |  |  |
| 2000−2011 | 939 (14.7%) | 5023 (12.6%) |
| 2012−2014 | 2534 (39.6%) | 16288 (41.0%) |
| 2015−2018 | 2920 (45.7%) | 18462 (46.4%) |
| **Alcohol intake history** |  |  |
| No | 5376 (84.1%) | 32443 (81.5%) |
| Yes | 1017 (15.9%) | 7330 (18.4%) |
| **Smoking and alcoholism history** |  |  |
| Never smoker | 5218 (81.6%) | 30236 (76.0%) |
| Non-drinker smokers | 537 (8.4%) | 4164 (10.5%) |
| Drinker-smokers | 638 (10.0%) | 5373 (13.5%) |
| **Family history** |  |  |
| No | 5584 (87.3%) | 36318 (91.3%) |
| Yes | 809 (12.7%) | 3455 (8.7%) |
| **Metastasis at diagnosis** |  |  |
| M0 | 2432 (38.0%) | 13091 (32.9%) |
| M1 | 1464 (22.9%) | 7103 (17.9%) |
| Unknown | 2497 (39.1%) | 19579 (49.2%) |

**Supplementary Table 4. Demographic and clinical characteristics of the cohort M (n = 2 272)**

| **Variable** | **Cohort M** |
| --- | --- |
|  |  |
| **Age, years** |  |
| 18−39 | 286 (12.6%) |
| 40−59 | 1121 (49.3%) |
| 60−79 | 838 (36.9%) |
| ≥ 80 | 27 (1.2%) |
| **Sex** |  |
| Male | 1394 (61.4%) |
| Female | 878 (38.6%) |
| **Year of diagnosis** |  |
| 2000−2011 | 239 (10.5%) |
| 2012−2014 | 868 (38.2%) |
| 2015−2018 | 1165 (51.3%) |
| **Primary tumor location** |  |
| Cecum, ascending and transverse colon cancer | 506 (22.3%) |
| Splenic flexure, descending and sigmoid colon cancer | 874 (38.5%) |
| Rectum | 892 (39.3%) |
| **Primary tumor sidedness** |  |
| Right-sided | 506 (22.3%) |
| Left-sided | 1766 (77.7%) |
| **History of cigarette smoking** |  |
| No | 1652 (72.7%) |
| Yes | 620 (27.3%) |
| **Alcohol intake history** |  |
| No | 1743 (76.7%) |
| Yes | 529 (23.3%) |
| **Family history** |  |
| Yes | 596 (26.2%) |
| No | 1676 (73.8%) |
| **Target therapy** |  |
| Cetuximab | 237 (10.4%) |
| Bevacizumab | 492 (21.7%) |
| No/Unknown | 1543 (67.9%) |

**Supplementary Table 5. Survival statistics of patients with metastatic colorectal cancer tumor regarding sidedness and history of smoking (n = 2 272)**

| **Patients** | **Median OS (95% CI)/Months** | **5-year OS** | ***P* value** | | | |
| --- | --- | --- | --- | --- | --- | --- |
|  |  |  | NS_R | ES_R | NS_L | ES_L |
| NS_R | 32.10 (24.62, 39.58) | 33.6% |  | 0.183 | 0.003 | 0.057 |
| ES_R | 34.87 (26.15, 43.59) | 34.9% | 0.183 |  | 0.985 | 0.677 |
| NS_L | 39.87 (35.17, 44.57) | 38.4% | 0.003 | 0.985 |  | 0.401 |
| ES_L | 33.79 (33.79, 41.81) | 35.4% | 0.057 | 0.677 | 0.401 |  |
| **Patients** | **HR (95% CI)** | | ***P* value** | | | |
| NS_R | Reference | |  | | | |
| ES_R | 0.78 (0.57, 1.06) | | 0.110 | | | |
| NS_L | 0.79 (0.67, 0.92) | | 0.003 | | | |
| ES_L | 0.85 (0.71, 1.01) | | 0.071 | | | |

Abbreviations: NS_R, never-smokers with right-sided colon cancer; ES_R, ever-smokers with right-sided colon cancer; NS_L, never-smokers with left-sided colorectal cancer; ES_L, ever-smokers with left-sided colorectal cancer.

**Supplementary Table 6. Death risks of cohort M regarding cigarette smoking on a quantitation basis ^a^**

|  | **All** | | **RSCC** | | **LSCRC** | |
| --- | --- | --- | --- | --- | --- | --- |
|  | **HR (95% CI)** | ***P* value** | **HR (95% CI)** | ***P* value** | **HR (95% CI)** | ***P* value** |
| **Number of Cigarettes per Day** |  |  |  |  |  |  |
| 0 | Reference |  | Reference |  | Reference |  |
| >0−10 | 0.97 (0.74, 1.28) | 0.847 | 0.87 (0.46, 1.64) | 0.660 | 1.02 (0.78, 1.39) | 0.905 |
| 11−20 | 1.06 (0.89, 1.27) | 0.523 | 0.73 (0.45, 1.16) | 0.185 | 1.16 (0.96, 1.42) | 0.133 |
| ≥21 | 0.98 (0.72, 1.34) | 0.916 | 0.81 (0.38, 1.72) | 0.578 | 1.04 (0.74, 1.45) | 0.840 |
| **Duration of Smoking (Years)** |  |  |  |  |  |  |
| 0 | Reference |  | Reference |  | Reference |  |
| >0−15 | 1.10 (0.84, 1.44) | 0.476 | 0.85 (0.51, 1.44) | 0.555 | 1.24 (0.92, 1.70) | 0.168 |
| 16−30 | 0.95 (0.79, 1.14) | 0.576 | 0.79 (0.47, 1.34) | 0.389 | 0.98 (0.80, 1.21) | 0.984 |
| >30 | 1.24 (0.97, 1.59) | 0.084 | 0.97 (0.50, 1.89) | 0.930 | 1.28 (0.98, 1.67) | 0.072 |
| **Smoking Index** |  |  |  |  |  |  |
| 0 | Reference |  | Reference |  | Reference |  |
| >0−600 | 0.95 (0.77, 1.17) | 0.629 | 0.88 (0.56, 1.38) | 0.587 | 0.98 (0.77, 1.25) | 0.982 |
| >600 | 1.13 (0.94, 1.37) | 0.198 | 0.77 (0.45, 1.33) | 0.354 | 1.22 (0.99, 1.49) | 0.059 |

**^a^** Analyses excluded corresponding missing cases.
